# Hem-O-Lok clip: a neglected cause of severe bladder neck contracture and consequent urinary incontinence after robot-assisted laparoscopic radical prostatectomy

**DOI:** 10.1186/1471-2490-14-21

**Published:** 2014-02-20

**Authors:** Luigi Cormio, Paolo Massenio, Giuseppe Lucarelli, Giuseppe Di Fino, Oscar Selvaggio, Salvatore Micali, Giuseppe Carrieri

**Affiliations:** 1 Department of Urology and Renal Transplantation, University of Foggia, viale Luigi Pinto 1, 71121 Foggia, Italy; 2 Department of Urology, University of Modena and Reggio Emilia, via del Pozzo 71, 41100 Modena, Italy

**Keywords:** Laparoscopy, Complications, Prostatectomy, Foreign body

## Abstract

**Background:**

Hem-o-lok clips are widely used during robot-assisted and laparoscopic radical prostatectomy to control the lateral pedicles. There are a few reports of hem-o-lok clip migration into the bladder or vesico-urethral anastomosis and only four cases of hem-o-lok clip migration resulting into bladder neck contracture. Herein, we describe the first case, to our knowledge, of hem-o-lok clip migration leading to severe bladder neck contracture and subsequent stress urinary incontinence.

**Case presentation:**

A 62-year-old Caucasian man underwent robot-assisted laparoscopic radical prostatectomy for a T1c Gleason 8 prostate cancer. One month after surgery the patient was fully continent; however, three months later, he presented with acute urinary retention requiring suprapubic drainage. Urethroscopy showed a hem-o-lok clip strongly attached to the area between the vesico-urethral anastomosis and the urethral sphincter and a severe bladder neck contracture behind it. Following cold-knife urethral incision and clip removal, the bladder neck contracture was widely resected. At 3-month follow-up, the patient voided spontaneously with a peak flow rate of 9.5 ml/sec and absence of post-void residual urine, but leaked 240 ml urine at the 24-hour pad test. To date, at 1-year follow-up, his voiding situation remains unchanged.

**Conclusions:**

The present report provides further evidence for the risk of hem-o-lok clip migration causing bladder neck contracture, and is the first to demonstrate the potential of such complication to result into stress urinary incontinence.

## Background

Hem-o-lok® clips (Weck Surgical Instruments, Teleflex Medical, Durham, NC) are widely used during laparoscopic radical prostatectomy for controlling the lateral pedicles. Their migration into the urinary tract has already been described [1-7], presenting with spontaneous expulsion, urethral erosion, bladder stone formation and even bladder neck contracture (BNC). There is no previous report of stress urinary incontinence (SUI) following removal of a migrated hem-o-lok clip. Herein we present the first case of hem-o-lok clip migration into the vesico-urethral anastomosis leading to severe bladder neck contracture (BNC) and subsequent SUI.

## Case presentation

**Figure 1:**
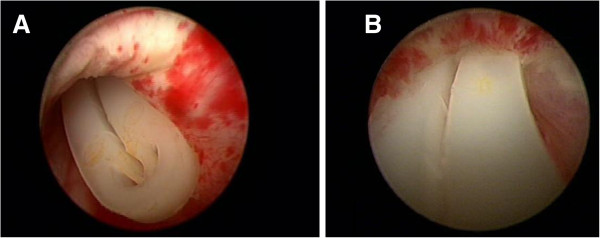
**Hem-o-lok clip within the lumen of the urethra*.*** Urethroscopy showing a hem-o-lok clip within the lumen of the urethra between the proximal part of the urethral sphincter and the distal part of the vesico-urethral anastomosis. The urethral sphincter looks functional though its white appearance at 12-o-clock position suggests some fibrotic reaction to the clip **(A)**; conversely, the distal part of the vesico-urethral anastomosis looks strictured over the clip **(B)**.

A 62-year-old Caucasian man underwent robot-assisted laparoscopic radical prostatectomy (RALP) because of a T1c Gleason 4 + 4 prostate cancer. Preoperative bone scan was negative. The surgical procedure and the postoperative course were both uneventful. At one-month follow-up, the patient was fully continent; pathology showed a pT3bN0 Gleason 8 (4 + 4) prostate cancer with negative surgical margins. Serum prostatic specific antigen (PSA) was 0.003 ng/mL. He was scheduled for close follow-up with serum PSA every 3 months. Three months after surgery, however, he presented with acute urinary retention requiring suprapubic drainage. Urethroscopy showed a hem-o-lok clip within the lumen of the urethra between the proximal part of the urethral sphincter and distal part of the vesico-urethral anastomosis (Figure 1). The urethral sphincter looked functional but its white appearance at 12-o-clock position suggested some fibrotic reaction to the presence of the clip (Figure 1A); conversely, the distal part of the vesico-urethral anastomosis looked strictured over the clip (Figure 1B).

**Figure 2:**
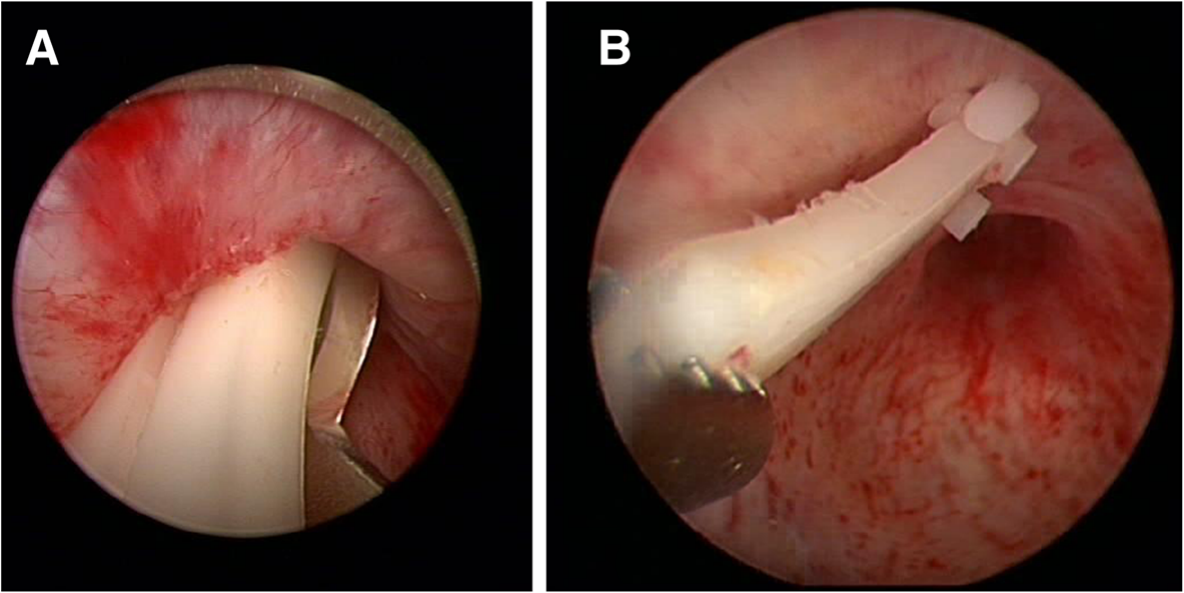
**First step treatment*.*** Cold-knife incision of the distal part of the contractured vesico-urethral anastomosis **(A)** and subsequent clip removal with a forceps **(B)**.

**Figure 3:**
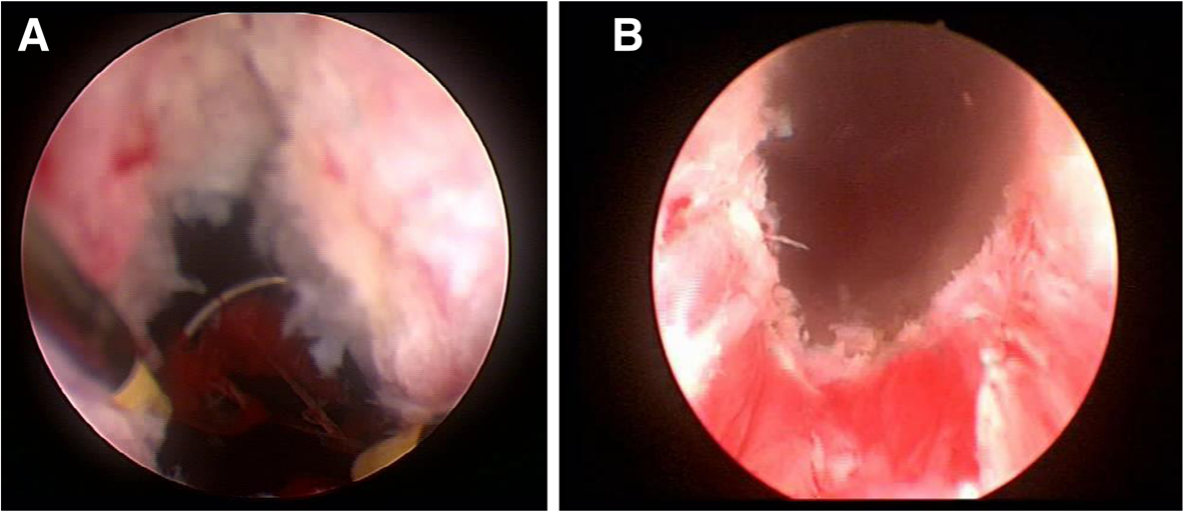
**Second step treatment*.*** Resection of the contractured bladder neck **(A)**, at the end, the urethral sphincter continued to look functional **(B)**.

As a matter of fact, an attempt to remove the clip with a forceps failed making cold-knife incision of the distal part of the contractured vesico-urethral anastomosis (Figure 2A) necessary to remove the clip with a forceps (Figure 2B). This maneuver exposed a severe BNC that had to be resected to access the bladder (Figure 3A); at the end of bladder neck resection the urethral sphincter continued to look functional (Figure 3B). At catheter removal, on second postoperative day, the patient voided spontaneously, had no post-void residual urine, but leaked some urine. At 3-month follow-up, the patient voided spontaneously with a peak flow rate of 9.5 ml/sec and absence of post-void residual urine, but leaked 240 ml urine at the 24-hour pad test. To date, at 1-year follow-up, his voiding situation remains unchanged.

The vesico-urethral anastomosis represents a potential site for clip migration. Palou et al. [8] reported migration of a metal clip after retropubic radical prostatectomy (RRP) presenting with perineal pain; Long et al. [9] reported migration of a metal clip after RRP causing BNC. Yi et al. [10] recently reported 4 further cases of metal clip migration after RRP; two resulted in stone formation and the other two into BNC. There are few reports of hem-o-lok clip migration into the urinary tract leading to spontaneous expulsion, urethral erosion, bladder stone formation and bladder neck contracture [1-7]. These findings would suggest that the use of clips in proximity of the vesico-urethral anastomosis should be limited if not avoided, and that the occurrence of *de novo* voiding symptoms in patients having undergone radical prostatectomy should rise the suspect of clip-related complications. Specifically, our patient was continent one month after surgery but developed *de novo* urinary retention two months later.

BNC represents an uncommon yet unpleasant clip-related complication. BNC following hem-o-lok clip migration was first described by Blumenthal et al [1]; in their series of 524 RALPs, 2 patients (0.4%) had a hem-o-lok clip-related BNC. The first, dealt with clip removal and KTP laser vaporization of the stricture, ended up to be continent but on self-catheterization to prevent stricture recurrence; the second, dealt with clip removal and transurethral incision with steroid injection, also ended up on self catheterization to prevent stricture recurrence yet used 1 pad/day for an undefined form of incontinence. More recently, Yi et al. [10] reported hem-o-lok clip-related BNC in 2 (1.3%) of the 153 patients they treated with RALP. Both cases were successfully managed by clip removal and a single urethral dilatation; both did not require self-catheterization and ended up in no recurrence nor urinary leak.

Our case had to be dealt with cold-knife urethral incision to remove the clip, and wide bladder neck resection. It is difficult to establish whether incontinence was due to a too large resection of the stricture causing an accidental injure to the sphincter or the sphincteric function having been jeopardised by a fibrotic reaction to the presence of the clip itself, as suggested by the whitish appearance of the urethral sphincter at 12-o-clock position. Whatever the case, our patient did not require self catheterization to prevent recurrence but developed urinary incontinence that was made even more bothersome by the fact that he had regained continence already one month after radical prostatectomy.

## Conclusions

The present case provides further evidence for the risk of hem-o-lok clip migration causing BNC and is the first to demonstrate the potential of such complication to result into stress urinary incontinence with its unpleasant consequences.

## Consent

Written informed consent was obtained from the patient for publication of this Case report and any accompanying images. A copy of the written consent is available for review by the Editor of this journal.

## Competing interests

The authors declare no potential competing interests.

## Authors’ contributions

LC: conception and manuscript revision. PM: data acquisition. GL: data analysis. GD: data analysis. OS: manuscript drafting. SM: data acquisition and manuscript revision. GC: supervision. All authors read and approved the final manuscript.

## Pre-publication history

The pre-publication history for this paper can be accessed here:

http://www.biomedcentral.com/1471-2490/14/21/prepub
